# Single-Nucleus RNA Sequencing Reveals the Transcriptome Profiling of Ovarian Cells in Adolescent *Cyprinus carpio*

**DOI:** 10.3390/ani14223263

**Published:** 2024-11-13

**Authors:** Mingxi Hou, Jin Zhang, Qi Wang, Ran Zhao, Yiming Cao, Yingjie Chen, Kaikuo Wang, Ning Ding, Yingjie Qi, Xiaoqing Sun, Yan Zhang, Jiongtang Li

**Affiliations:** 1 Key Laboratory of Aquatic Genomics, Ministry of Agriculture and Rural Affairs, Beijing Key Laboratory of Fishery Biotechnology, Chinese Academy of Fishery Sciences, Beijing 100141, China; houmingxi@cafs.ac.cn (M.H.); zhangjin@cafs.ac.cn (J.Z.); wangqi@cafs.ac.cn (Q.W.); zhaoran@cafs.ac.cn (R.Z.); caoyiming@cafs.ac.cn (Y.C.); 18842022137@163.com (N.D.);sunxiaoqing@cafs.ac.cn (X.S.); zhangyan@cafs.ac.cn (Y.Z.); 2 National Demonstration Center for Experimental Fisheries Science Education, Shanghai Ocean University, Shanghai 201306, China; cyjttkl@163.com (Y.C.); 18631836881@163.com (K.W.); qyj521720@163.com (Y.Q.); 3 Chinese Academy of Agricultural Sciences, Beijing 100081, China

**Keywords:** common carp, single-nucleus sequencing, gene expression, oogenesis, granulosa cell

## Abstract

The common carp (*Cyprinus carpio*) is a crucial freshwater species cultivated worldwide for food consumption. Female carp have better growth performance than males, which fascinates scholars to uncover the mechanism of gonadal differentiation and produce mono-sex populations. However, the mechanism of ovarian development at single-cell resolution is limited. Here, we conducted single-nucleus RNA sequencing in adolescent common carp ovaries. Our study obtained transcriptional profiles of 13,155 nuclei and revealed 13 distinct cell clusters in the ovaries, including three subtypes of germ cells and four subtypes of granulosa cells. We subsequently performed pseudotime trajectory analysis to delineate potential mechanisms underlying the development of germ cells and granulosa cells. We identified 1250 dynamic expression genes in germ cells and 1815 in granulosa cells (*q*-value < 0.01), including *zp3*, *eif4a2* and *aspm* in germ cells and *fshr* and *esr1* in granulosa cells. The functional annotation showed that dynamic expression genes in germ cells were involved in sperm–egg recognition and some terms related to meiosis, such as sister chromatid segregation and homologous recombination. Genes expressed dynamically in granulosa cells were related to the TGF-β signaling pathway, response to gonadotropin, and development of primary female sexual characteristics. In addition, the dynamic genes expressed in granulosa cells might relate to the complex communication between different cell types. In summary, our study provided a transcriptome profile of common carp ovaries at single-nucleus resolution, and we further revealed the potential cell type-specific mechanisms underlying oogenesis and the differentiation of granulosa cells, which will facilitate breeding all-female common carp populations.

## Simple Summary

The common carp (*Cyprinus carpio*) shows pronounced sexual dimorphism in growth, and females grow faster than males. Studying the molecular mechanisms of ovarian development and oogenesis is vital for improving productivity and breeding all-female populations. Although many transcriptome analyses of bulk ovaries have been conducted, they have not been able to uncover cell type-specific differences in gene expression. In this study, we presented the transcriptional profile of common carp ovaries at the single-cell level using single-nucleus RNA sequencing. Our analysis revealed valuable information about regulating oogenesis and the development of granulosa cells. Our research provides a helpful resource that complements previously published bulk ovary transcriptomic analyses in the common carp for further investigating the molecular and cellular mechanisms underlying ovarian development.

## 1. Introduction

The common carp is one of the most critical cyprinid species with an XX/XY sex-determining system, providing a good model for studying gonadal differentiation in cyprinidae fish [1]. Female common carp exhibit faster growth rates than males, and breeding an all-female population can generate higher economic benefits [2]. Research on the gonadal development of common carp has important implications for improving productivity and sustainable production.

Ovarian development and oogenesis are the basis of sexual reproduction [3]. In teleost, the ovary arises from the genital ridge, which displays the potential for bi-directional differentiation and is composed of primordial germ cells and uncharacterized somatic precursor cells [4,5]. Primordial germ cells differentiate into oogonia, and oogonia proliferate inside cysts via mitosis and then undergo meiosis to develop into oocytes during ovarian development. Oocytes eventually undergo continuous growth and the deposition of yolk and finally form mature eggs [6,7,8]. The somatic precursor cells will differentiate into granulosa cells, theca cells, and epithelial cells [9,10]. Granulosa cells, also known as supporting cells, enclose around germ cells directly, and theca cells are located in the mesenchyme [11]. Theca cells can produce androgens, which are subsequently converted to estrogens by granulosa cells [12]. In addition, other cell types are present in the ovary, such as T cells and stromal cells [13].

The gene expression levels vary dramatically across different cell types in the ovary. Previous studies have identified many marker genes of germ cells, such as *vasa* and *piwil* [14,15]. *sycp3* is expressed only in meiotic oocytes [16]. In ovarian somatic cells, *foxl2* and *wnt1* are expressed explicitly in granulosa cells [17], while *star* and *cyp17a1* are highly expressed in theca cells [18]. Not only do different cell types have significant differences in gene expression, but the same cell type at different developmental stages also displays different gene expression patterns [13,19]. Dissecting the gene expression patterns of different cell types is critical for further understanding the molecular and cellular mechanisms of oogenesis and ovarian differentiation.

Previous studies have revealed histological and molecular alterations during ovarian development of the common carp [20,21]. Oogonia undergo meiotic division at 45 days post-hatch (dph), and oocytes in meiosis first appear at 55 dph. At about 120 dph, germ cells at different stages fill the whole ovarian cavity. Many genes, such as *foxl2*, *cyp19a1a*, *sycp3*, and *dmc1*, show dynamic expression during ovarian development [21]. Although some transcriptome profiles have investigated differential gene expression among different ovarian stages [22,23], the conventional bulk transcriptomic assays on the whole ovary only reflect the average gene expression across the diverse constituent cell types. There is still much room to study the mechanisms underlying the ovarian development of common carp at single-cell resolution.

In recent years, single-cell RNA sequencing (scRNA-seq) and single-nucleus RNA sequencing (snRNA-seq) have become powerful tools to clarify single-cell gene expression patterns and further elucidate physiological processes [24,25,26]. A challenge for scRNA-seq is the difficulty in capturing some cell types, like irregularly shaped or giant cells, while snRNA-seq has showed better performance in these situations [27,28,29].

In the present study, we conducted snRNA-seq to reveal the transcriptional profile of common carp ovaries at the cellular level, which complements prior published bulk transcriptomic analyses. Furthermore, we provided valuable information about the regulation mechanisms of oogenesis and the development of granulosa cells. Our data will be helpful for the further study of molecular and cellular mechanisms of ovarian development in the common carp.

## 2. Materials and Methods

### 2.1. Materials

Experimental animals were obtained from the Fangshan Experimental Station in Beijing, China. The adolescent common carp (four months old) was dissected, and the gonads on both sides of the abdominal cavity were used. One side of the gonads was fixed in Bouin’s solution overnight at 4 °C, while the other side was frozen in liquid nitrogen and then stored in a refrigerator at −80 °C. There were three biological duplicates in this study. All possible efforts were taken to minimize the suffering of the animals.

### 2.2. Hematoxylin and Eosin (H&E) Staining

The gonads fixed in Bouin’s solution were subjected to dehydration, penetration, and immersion in paraffin in an automatic dehydrator (KD-TS3A, Kedee, Jinhua, China). The procedures and parameters were as follows: 75% ethanol for 1 h, 85% ethanol for 1 h, 95% ethanol for 1 h, 100% ethanol I for 1 h, 100% ethanol II for 1 h, a mixture of xylene and 100% ethanol in a 1:1 ratio for 15 min, xylene I for 8 min, xylene II for 5 min, a mixture of xylene and paraffin in a 1:1 ratio for 30 min, paraffin I for 1 h, and paraffin II for 1 h. Samples were further embedded in paraffin and manually sectioned into 5 μm thick paraffin slices with a rotary microtome (KD-2258, Kedee). The sections were stained with hematoxylin and eosin (H&E) using the following procedure: xylene I for 1 min, xylene II for 1 min, 100% ethanol for 1 min, 95% ethanol for 1 min, 80% ethanol for 1 min, hematoxylin for 8 min, washing with running water for 1 min, 1% hydrochloric acid solution in ethanol for 15 s, 0.5% eosin for 30 s, 80% ethanol for 1 min, 95% ethanol for 1 min, 100% ethanol for 1 min, xylene I for 2 min, and xylene II for 2 min. Photomicrographs were collected using an automatic digital slide scanner (Aperio Versa 8, Leica, Wetzlar, Germany).

### 2.3. Library Preparation and Sequencing

We isolated single-cell nuclei following a published protocol [30]. The gonad tissues were pooled and ground in the pre-chilled Nuclei Extraction Buffer (Miltenyi Biotech, Bergisch Gladbach, Germany) until nuclei were fully released. Cell strainers (40 µm) were used to filter cell debris and clumps, and filtrates were collected in a new centrifuge tube. The nuclei were obtained at the bottom of the tube after centrifugation, and then they were washed and resuspended in the blocking buffer (0.07% BSA, 1× PBS, and 0.1% RNase inhibitor). The library of snRNA-seq was prepared using the Chromium Single Cell 3′ Reagent Kits v3 (10x Genomics, Pleasanton, CA, USA). Briefly, the nucleus suspension was mixed with the barcoded Gel Beads, reverse transcriptase, and partitioning oil to form the Gel Beads-In-Emulsion. After finishing reverse transcription, the emulsions were broken, and the barcoded cDNA was purified with magnetic beads. Then, PCR amplification was performed to construct the 3′ gene expression library. The snRNA-seq library was sequenced on a DNBSEQ-T7 platform.

### 2.4. Read Filtering and Alignment

The raw data of fastq files were filtered to remove adapter sequences, reads containing more than three Ns, and low-quality reads (the percentage of bases with quality values below five exceeding 20%) using FastQC (v0.11.3). The common carp reference genome and the transcript annotation GTF files were downloaded from NCBI [31]. Splicing-aware alignment of reads to the reference genome was performed using STAR in Cell Ranger (v7.1.0). Then, the mapped reads were bucketed into exonic, intronic, and intergenic reads according to the transcript annotation GTF files in Cell Ranger.

### 2.5. Cell Identification and Gene Expression Quantification

The cell-versus-gene expression matrix was generated in Cell Ranger. Briefly, the cell barcodes provided information about which cell the reads came from, and UMIs (unique molecular identifiers) were used to distinguish between PCR duplicates and transcript abundance. Reads that shared the same barcode and gene annotation were grouped, and the gene expression level was calculated based on the counts of unique UMIs.

### 2.6. Identification of Cell Types

The data underwent filtering again to obtain reliable and precise analytical outcomes. Genes detected in at least ten cells were deemed confidently expressed, and cells with expressed gene counts ranging from 500 to 7000 were retained in this study. After normalizing the gene expression levels using the “SCTransform” function in R package Seurat (v2.2.0) [32], 2000 highly variable genes in the dataset were selected. Principal component analysis (PCA) was conducted to reduce the dimensionalities of the expression matrix using the IRLBA algorithm [33]. Cells with similar gene expression patterns were classified into one cluster. The PCA-reduced data were visualized using t-stochastic neighbor embedding (t-SNE) in Cell Ranger [34]. The non-parametric Wilcoxon rank sum test was performed to identify differentially expressed genes among clusters. We used the COSG (cosine similarity-based marker gene identification) method to identify marker genes in every cluster [35]. Cell clusters were annotated according to the function of marker genes and differentially expressed genes.

### 2.7. Construction of Pseudotime Trajectories

Single-cell pseudotime trajectory analysis can clarify the direction of cell differentiation and excavate intermediate cell states and key regulators during cell differentiation [36]. We conducted pseudotime trajectory analysis for germ cells and granulosa cells using Monocle3 (v1.2.9) [36]. The matrix of cell-versus-gene expression was the input, and Monocle3 reduced the space to two dimensions and ordered the cells with default parameters. Dynamic genes during cell differentiation were identified using the ‘differentialGeneTest’ function, with a parameter of *q*-value < 0.01.

### 2.8. GO and KEGG Pathway Enrichment

Gene Ontology (GO) and Kyoto Encyclopedia of Genes and Genomes (KEGG) enrichment analyses of dynamic expression gene sets were implemented in the ClusterProfiler R package (v3.9) [37]. Significantly enriched terms were those with an adjusted *p*-value < 0.05.

## 3. Results

### 3.1. Major Cell Types in Adolescent Common Carp Ovaries

**Figure 1:**
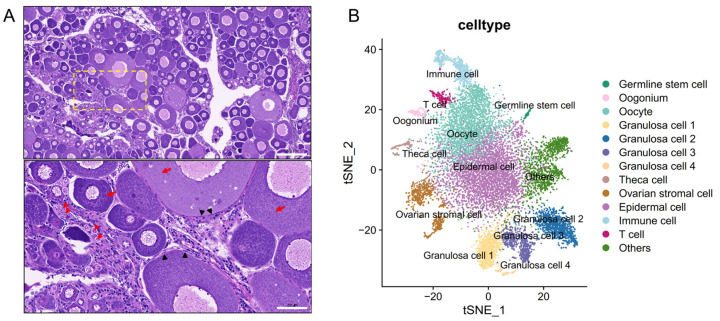
Cell types in the common carp ovary. (**A**) Observation of histological features of major cell types in the ovary using H&E staining. Scale bar = 200 µm (top), 50 µm (bottom); red arrows, oocyte; red arrowheads, oogonia; black arrowheads, granulosa cell. (**B**) Visualization of snRNA-seq data in t-SNE to reveal distinct cell clusters.

We assessed the cell types histologically according to previously published documents [9,38]. The germ cells at different stages were observed in the ovary. Oogonia reside within the ovarian germinal epithelium, characterized by a relatively large nucleus and minimal cytoplasm. Oogonia can self-renew, and some of them differentiate into the growing oocytes. During oocyte growth, nucleoli emerge at the periphery of the enlarged nucleus while nutrient reserves are gradually increased in the cytoplasm. The follicle, the functional unit of the ovary, generally refers to an oocyte plus its surrounding cell sheath. Granulosa cells, one layer of flattened cells, are directly enclosed around the oocytes, and the outside layer consists of theca cells and other cell types. The histological features of the adolescent common carp ovary in H&E staining are shown in Figure 1A.

### 3.2. Overview of Common Carp Ovary Single-Nucleus Transcriptome Sequencing

We conducted ovary single-nucleus sequencing and obtained 77.65 G of raw data, which we deposited into the NCBI BioProject database under the accession number PRJNA1065616. snRNA-seq included a total of 13,849 nuclei, and 47,775 genes were detected. After quality filtering, 13,155 nuclei were retained for subsequent analyses. These nuclei were classified into 13 cell clusters, and the differentially expressed genes in each cluster were analyzed (Table S1). There were 378 differentially expressed genes across cell clusters under the threshold log_2_-fold change ≥ 1 and corrected *p*-value < 0.05 (false discovery rate corrected).

### 3.3. Identification of Cell Types in Common Carp Ovaries

We calculated the top 10 marker genes in each cluster using the COSG method (Table S2). According to the function of marker genes and differentially expressed genes in each cluster, cell clusters were annotated and visualized in a t-SNE representation (Figure 1B). The germ cells were identified by the expression of *zp3*, which is essential for the zona pellucida [39], while *rad50* was mainly expressed in mitotic germ cells [25]. *Eif4a1b* and *eif4a2* were involved in female germ cell self-renewal and asymmetric division [40]. Three clusters of germ cells, defined as germ stem cells, oogonia, and oocytes, were identified. Granulosa cell markers, such as *fshr*, *notch1*, *wt1*, *gsdf*, and *bmpr2*, were expressed in four clusters [13,17]. *Tgf-α*, which promotes androgen biosynthesis, was expressed in theca cells [11]. We finally obtained 3072 (23.35%) germ cells and 2645 (20.11%) granulosa cells, and the proportions of the major cell types in the ovary are shown in Table 1. We focused on germ cells and granulosa cells for subsequent analysis.

### 3.4. Trajectory Analyses of Germ Cells and Granulosa Cells

**Figure 2:**
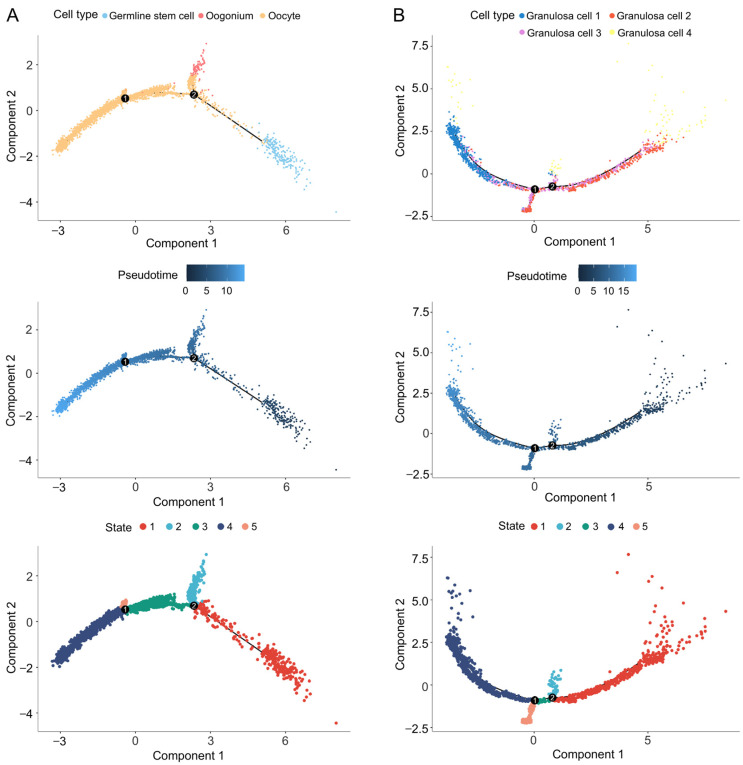
The pseudotime trajectory analyses. (**A**) Differentiation trajectories of germ cells. (**B**) Differentiation trajectories of granulosa cells. The numbers inside the black circles represent the branch points of different cell states; different colors in the top two figures represent different cell types; the colors from dark to light in the middle two figures represent the degree of differentiation; different colors in the bottom two figures represent different cell states.

Oogenesis is a dynamic and orchestrated process during ovarian development. We conducted pseudotemporal ordering analysis of the germ cell subtypes to elucidate the molecular basis of oogenesis. We defined germline stem cells as the initial stage and oocytes as the ultimate stage. Three germ cell subtypes showed a linear relationship along differentiation trajectories, and five cell states were distinguished (Figure 2A). The granulosa cells are another critical cell group in the ovary; we also conducted differentiation trajectories of granulosa cells, as shown in Figure 2B.

### 3.5. Gene Expression Dynamics During the Differentiation of Germ Cells and Granulosa Cells

We analyzed gene expression patterns during cell differentiation. There were 1250 genes in germ cells and 1815 genes in granulosa cells that displayed dynamic expression changes under a threshold of *q*-value < 0.01 (Tables S3 and S4). Some well-known genes essential for the development of oocytes, such as *zp3*, *eif4a2*, and *aspm*, showed dynamic changes across different germ cells (Table S3). Meanwhile, genes (such as *fshr* and *esr1*) related to hormone pathways were dynamically expressed in different granulosa cell subtypes (Table S4).

### 3.6. Functional Analysis of Dynamic Expression Genes

**Figure 3:**
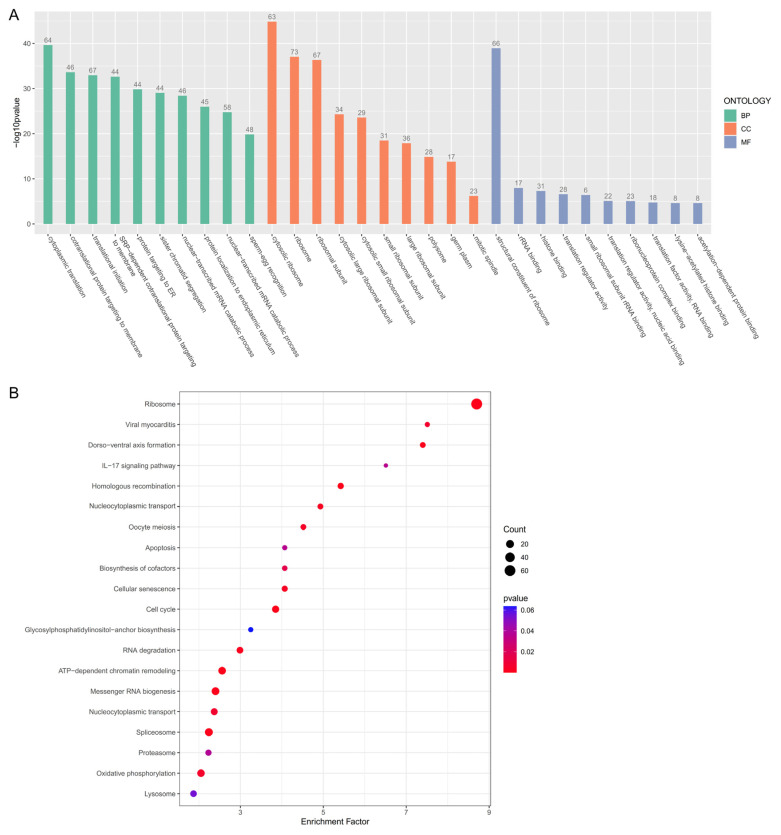
Functional annotation of dynamic expression genes in germ cells. (**A**) The bar plot of GO enrichment analysis of dynamic expression genes in germ cells. The number on the bar means gene numbers enriched in this term. BP, biological processes; CC, cell component; MF, molecular function. (**B**) The bubble diagram of KEGG enrichment analysis of dynamic expression genes in germ cells. The greater the enrichment factor, the more reliable the significance of differential gene enrichment in this pathway.

These dynamic expression genes were subjected to enrichment analysis. The GO analysis revealed that the dynamic expression genes in germ cells participated in translational initiation, sister chromatid segregation, and sperm–egg recognition at the biological processes level; many terms related to the ribosome and germ plasm at the cell component level; and histone binding and nucleic acid binding at the molecular function level (Figure 3A). The KEGG analysis showed that the dynamic genes in germ cells were significantly enriched for homologous recombination, oocyte meiosis, and the biosynthesis of cofactors (Figure 3B).

**Figure 4:**
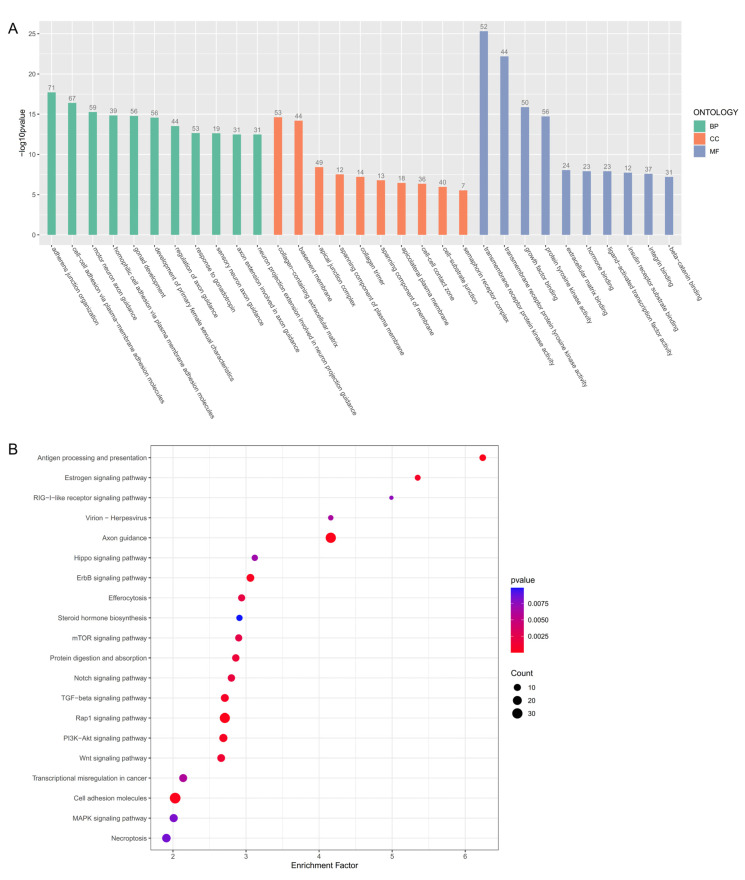
Functional annotation of dynamic expression genes in granulosa cells. (**A**) The bar plot of GO enrichment analysis of dynamic expression genes in granulosa cells. The number on the bar means gene numbers enriched in this term. BP, biological processes; CC, cell component; MF, molecular function. (**B**) The bubble diagram of KEGG enrichment analysis of dynamic expression genes in granulosa cells. The greater the enrichment factor, the more reliable the significance of differential gene enrichment in this pathway.

During the differentiation of granulosa cells, the GO analysis showed that dynamically expressed genes were involved in the response to gonadotropin, the development of primary female sexual characteristics, hormone binding, and the cell–cell contact zone (Figure 4A). The KEGG enrichment revealed that dynamic genes were related to the estrogen signaling pathway, steroid hormone biosynthesis, the TGF-β signaling pathway, and cell adhesion molecules (Figure 4B).

## 4. Discussion

Despite many studies attempting to elucidate the molecular mechanisms underlying gonadal differentiation of the common carp [21,22,23], the mechanisms of ovarian development at the single-cell level remain poorly understood. According to the histological analysis, the ovary of adolescent common carp is a highly heterogeneous organ containing germ cells at different stages and various types of somatic cells. In *Cynoglossus semilaevis* and *Lates calcarifer*, scRNA-seq were conducted to reveal the mechanisms of ovarian development [25,41]. In teleost, oocytes are fragile, and their size varies enormously, which may result in the loss of some cell types in tissue dissociation and cell filtration during scRNA-seq. snRNA-seq may be a better choice in this situation. Here, the single-nucleus analysis included 13,155 nuclei and 47,775 genes. To our knowledge, this is the first ovary single-nucleus transcriptional profile in teleost.

Although snRNA-seq does not include RNA molecules from the cytoplasm, many studies have shown that the transcriptional profiles obtained from snRNA-seq can reflect the transcriptional status of different cell types and are comparable to those obtained from scRNA-seq [42,43,44]. In this study, we identified 13 distinct cell clusters in the ovary of adolescent common carp; among them, the number of epidermal cells (31.91%), germ cells (23.35%), and granulosa cells (20.11%) accounted for a large proportion. All the major cell types in ovaries were contained in this transcriptional profile, indicating that the data provided here are reliable and can be used as a reference for further studies related to common carp ovarian development.

Some well-known genes, such as *dnd1*, *vasa*, and *nanos3*, did not exhibit high expression in germ cells in the present transcriptional profile compared with previous studies [25,41]. *dnd1* is essential for the migration and survival of primordial germ cells [45]. *Vasa* and *nanos3*, highly expressed in the cytoplasm, were often used as the marker genes of germ cells [5,41,46]. This discrepancy may be attributed to the continuous accumulation of their transcripts in the yolk during oogenesis and the fact that our results of snRNA-seq did not include transcripts from the yolk.

We further conducted pseudotime trajectory analyses to explore the dynamic molecular regulation during oogenesis and the development of granulosa cells. Germ cells undergo mitosis for self-renewal and proliferation and then meiosis to differentiate into oocytes [6,7,8]. Here, we identified three germ cell subtypes (germ stem cells, oogonia, and oocytes), and they showed a linear developmental relationship. The expression of 1250 genes varied during oogenesis (*q*-value < 0.01). Among them, *aspm* is essential for replication fork stability during cell division [47], and *eif4a2* participates in germ cell self-renewal and asymmetric division [40]. The enrichment analyses showed that these dynamic expression genes were associated with meiosis (sister chromatid segregation and homologous recombination) and the maintenance of oocyte identity (sperm–egg recognition and germ plasm component). In addition, many terms related to the ribosome were enriched (Figure 3). The ribosome and ribosome-related genes might play vital roles in oogenesis.

The differentiation of granulosa cells occurs concurrently with follicular growth [8,17]. We identified 1815 dynamic expression genes across different subtypes of granulosa cells (*q*-value < 0.01). FSH (follicle-stimulating hormone), secreted by the pituitary gland, is responsible for the survival and proliferation of granulosa cells, but it is independent of their early development [48]. *fshr* (encoding the FSH receptor) showed dynamic expression in our analyses. The dynamic genes expressed in granulosa cells were enriched in the development of primary female sexual characteristics and pathways related to steroid hormones. These results suggest that the provided transcriptome profile and our analyses are reliable.

The complex communication between different cell types is essential to promote and maintain ovarian differentiation and oogenesis. In mammals, oocyte-derived GDF9 can regulate the expression of *Dhh* and *Ihh* in granulosa cells, which is essential for establishing theca cells [10]. The expression of *bmp15* in oocytes is associated with ovarian maintenance by promoting the development of granulosa cells and regulating estrogen biosynthesis in zebrafish [49], and estrogen can regulate feedback oogenesis [6]. The dynamic genes expressed in granulosa cells were enriched in the cell–cell contact zone, extracellular matrix, and cell adhesion molecules (Figure 4), which might relate to the complex communication between different cell types. The cell-to-cell crosstalk is worthy of further study to elucidate the mechanism of cell development and ovarian differentiation in the common carp.

## 5. Conclusions

In the present study, we conducted snRNA-seq in the ovaries of adolescent common carp. We identified 13 distinct cell clusters, including three clusters of germ cells and four clusters of granulosa cells. We then conducted pseudotime trajectory analyses of germ cells and granulosa cells and mined the dynamic expression genes during cell development. The functional annotation showed that dynamic expression genes in germ cells were involved in sperm–egg recognition and terms related to meiosis. Genes expressed dynamically in granulosa cells were related to the pathways of hormones and terms promoting cellular communication. Briefly, we provide an ovarian single-nucleus transcriptome profile and a comprehensive understanding of oogenesis and the development of granulosa cells in the common carp at single-cell resolution. Our study is a valuable resource that complements previously published bulk ovary transcriptomic analyses.

## Figures and Tables

**Table 1:** The proportion of cells in different clusters.

Clusters	Number of Cells		Proportion of Clusters	
Germline stem cell	88	3072	0.67%	23.35%
Oogonium	192		1.46%	
Oocyte	2792		21.22%	
Granulosa cell 1	827	2645	6.29%	20.11%
Granulosa cell 2	1049		7.97%	
Granulosa cell 3	678		5.15%	
Granulosa cell 4	91		0.69%	
Theca cell	180		1.36%	
Ovarian stromal cell	813		6.18%	
Epidermal cell	4198		31.91%	
Immune cell	644		4.90%	
T cell	235		1.79%	
Others	1368		10.40%	
Total	13,155		100%	

## Data Availability

The data of snRNA-seq presented in the current study have been submitted to the NCBI BioProject database under the accession number PRJNA1065616.

## Supplementary Materials

The following supporting information can be downloaded at: https://www.mdpi.com/article/10.3390/ani14223263/s1. Table S1: the differentially expressed genes among different clusters; Table S2: the top 10 marker genes in each cluster using the COSG method; Table S3: Dynamic expression genes during germ cell development; Table S4: Dynamic expression genes during granulosa cell development.

## Abbreviations

scRNA-seq, single-cell RNA sequencing; snRNA-seq, single-nucleus RNA sequencing; dph, days post-hatch; H&E staining, hematoxylin and eosin staining; UMIs, unique molecular identifiers; PCA, principal component analysis; t-SNE, t-stochastic neighbor embedding; COSG, cosine similarity-based marker gene identification; GO, Gene Ontology; KEGG, Kyoto Encyclopedia of Genes and Genomes.
